# Prolonged and Refractory Hypophosphataemia Following Intravenous Ferric Carboxymaltose Administration Delaying Hospital Discharge: A Case Report

**DOI:** 10.7759/cureus.96659

**Published:** 2025-11-12

**Authors:** Zohaib Sajid

**Affiliations:** 1 Internal Medicine - Gastroenterology, University Hospitals Birmingham NHS Foundation Trust, Birmingham, GBR

**Keywords:** ferric carboxymaltose, hospital discharge delay, intravenous iron replacement, iron deficiency anaemia, iron-induced hypophosphataemia

## Abstract

Hypophosphataemia is an under-recognised complication of intravenous ferric carboxymaltose (Ferinject). While often transient and asymptomatic, in some cases, it may be severe and prolonged. We describe a man in his 40s with iron deficiency anaemia who received two 1,000 mg doses of ferric carboxymaltose within one week. Despite normal baseline renal function and calcium, and only borderline vitamin D insufficiency, he developed profound hypophosphataemia with a nadir of 0.20 mmol/L. This required repeated intravenous phosphate replacement and delayed hospital discharge by eight days, despite the patient being otherwise medically fit. The case underscores that ferric carboxymaltose can cause clinically important hypophosphataemia, even in younger patients, and that routine monitoring should be considered.

## Introduction

Iron deficiency anaemia is a common clinical problem, affecting an estimated 1.2 billion people globally. While oral iron is usually the first-line therapy, intolerance, malabsorption, and lack of efficacy often necessitate intravenous replacement. Ferric carboxymaltose (Ferinject) is widely used due to its ability to rapidly correct iron deficiency with high single doses and a favourable infusion profile. However, hypophosphataemia has emerged as an important adverse effect, with studies by Wolf et al. and Schaefer et al. reporting incidences approaching 70% when defined as serum phosphate <0.8 mmol/L following ferric carboxymaltose therapy, compared with less than 10% in patients receiving ferric derisomaltose or iron sucrose [1-3].

This effect is mediated by increased intact fibroblast growth factor 23 (FGF23), which promotes renal phosphate wasting and suppresses 1α-hydroxylase, reducing intestinal absorption [4]. Although most cases are mild and transient, severe hypophosphataemia can lead to osteomalacia, fractures, myopathy, and arrhythmia [3]. Importantly, even in asymptomatic patients, biochemical hypophosphataemia can complicate care and delay hospital discharge, as demonstrated by Wolf et al. [1]. This case illustrates how ferric carboxymaltose-induced hypophosphataemia delayed discharge by over a week in an otherwise medically stable younger patient.

## Case presentation

A man in his 40s with a background of chronic pancreatitis, cirrhosis with portal vein cavernoma, oesophageal and gastric varices, and a previous gastrojejunostomy was referred by his general practitioner with symptomatic anaemia. He reported increasing fatigue and reduced exercise tolerance. Initial blood tests revealed haemoglobin of 60 g/L, ferritin of 12 µg/L, serum iron of 2.8 µmol/L, phosphate of 0.98 mmol/L, and vitamin D of 42.6 nmol/L. He had previously trialled oral iron therapy without adequate response. Given the urgency of correction and ineffectiveness of oral therapy, intravenous ferric carboxymaltose (Ferinject) was selected. *First admission (March-April 2024)*

He received two units of packed red blood cells, increasing haemoglobin to 79 g/L. Endoscopy demonstrated a large oesophageal ulcer with slow oozing, which was treated with adrenaline injection and high-dose IV omeprazole. He was administered intravenous Ferinject 1,000 mg on 28/03/24. Within one week, phosphate declined to 0.42 mmol/L, for which he received intravenous replacement. Despite transient improvement (0.58 mmol/L on 04/04/24), levels remained low (0.52 mmol/L on 05/04/24). A second 1,000 mg dose of Ferinject was given on 04/04/24 prior to discharge. He was discharged with oral supplements. *Second admission (April-May 2024)*

He re-presented on 24/04/24 with haemoglobin 69 g/L, which rose to 74 g/L without transfusion. Phosphate on 03/05/24 was 0.51 mmol/L, prompting intravenous replacement. On the same date, ferritin was 39 µg/L, confirming only partial iron repletion despite prior Ferinject doses. On 10/05/24, he was administered ferric derisomaltose to continue iron repletion while avoiding further hypophosphataemia risk. Values remained unstable throughout May, reaching a nadir of 0.20 mmol/L on 14/05/24 despite oral supplementation. He required multiple intravenous phosphate infusions (Table 1), but securing venous access was challenging and contributed to treatment delays; ultimately, central venous access was required. Repeat endoscopy on 17/05/24 showed Barrett’s mucosa with ulceration but no dysplasia or malignancy. Renal function remained normal (creatinine: 40-50 µmol/L, eGFR consistently >90 mL/min/1.73m²), with corrected calcium and magnesium stable, as detailed in Table 2. No alternative cause of hypophosphataemia was identified.

**Table 1:** Serum phosphate values and key interventions Values shown demonstrate the development of recurrent and severe hypophosphataemia following high-dose ferric carboxymaltose, requiring repeated intravenous phosphate replacement and contributing to delayed hospital discharge. Phosphate reference range: 0.80–1.50 mmol/L.

Date	Phosphate (mmol/L)	Intervention/Notes
28/03/24	0.98	Baseline (before first Ferinject)
03/04/24	0.42	Post Ferinject – IV phosphate given
04/04/24	0.58	Second Ferinject + IV phosphate
05/04/24	0.52	Persistently low
03/05/24	0.51	IV phosphate
05/05/24	0.65	Oral phosphate started
08/05/24	0.79	
09/05/24	0.76	
11/05/24	0.69	
13/05/24	0.43	On oral replacement
14/05/24	0.20	Severe nadir
15/05/24	0.54	IV phosphate
16/05/24	0.50	IV phosphate
17/05/24	0.44	IV phosphate
18/05/24	0.46	IV phosphate
19/05/24	0.65	
20/05/24	0.53	
21/05/24	0.52	IV phosphate
22/05/24	0.33	
23/05/24	0.30	IV phosphate
24/05/24	0.27	IV phosphate
25/05/24	0.50	IV phosphate
26/05/24	0.57	IV phosphate
27/05/24	0.49	IV phosphate
28/05/24	0.50	IV phosphate
29/05/24	0.50	
30/05/24	0.50	Discharge on oral phosphate

**Table 2:** Summary of additional biochemical results throughout admission Summary of additional biochemical parameters with observed ranges and reference intervals. Reference ranges are based on University Hospital Birmingham laboratory standards. Vitamin D levels showed borderline insufficiency. eGFR: estimated glomerular filtration rate; CRP: C-reactive protein

Parameter	Observed Range	Reference Range
Urea (mmol/L)	3.5–6.2	2.5–7.8
Creatinine (µmol/L)	40–50	45–120
eGFR (mL/min/1.73 m²)	>90	≥60
Sodium (mmol/L)	134–138	133–146
Potassium (mmol/L)	3.8–4.5	3.5–5.3
Corrected Calcium (mmol/L)	2.25–2.35	2.20–2.60
Magnesium (mmol/L)	0.78–0.87	0.70–1.00
Albumin (g/L)	36–42	35–50
CRP (mg/L)	<5	<5
Vitamin D (nmol/L)	42.6	≥50

**Figure 1:**
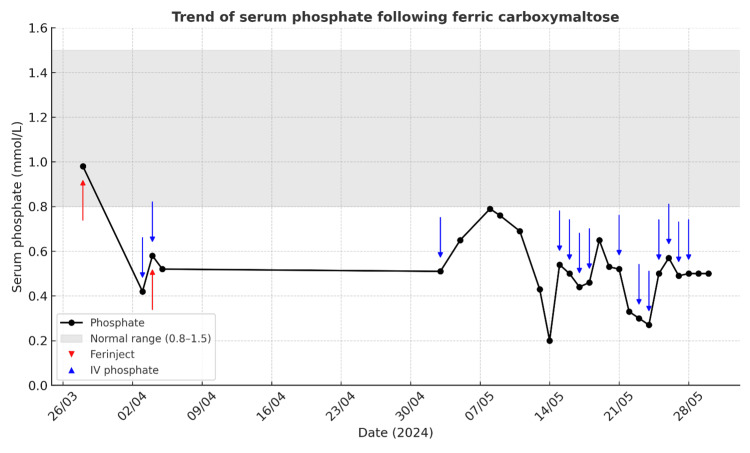
Trend of serum phosphate following ferric carboxymaltose administration The figure demonstrates prolonged and recurrent hypophosphataemia following two 1,000 mg doses of ferric carboxymaltose, with a nadir of 0.20 mmol/L and persistent instability despite repeated intravenous and oral phosphate supplementation. Phosphate reference range: 0.80–1.50 mmol/L.

By 22/05/24, he was clinically stable from a gastrointestinal perspective and otherwise fit for discharge, but refractory hypophosphataemia (<0.5 mmol/L) delayed discharge, as detailed in Figure 1. He remained inpatient for an additional eight days, during which further intravenous phosphate was given. He described frustration at being otherwise well yet kept in hospital, including having to spend his birthday as an inpatient. He was discharged on 30/05/24 with phosphate 0.50 mmol/L, on oral phosphate replacement, and remains on oral supplementation at outpatient follow-up.

## Discussion

Ferinject (ferric carboxymaltose) is one of the most commonly used intravenous iron formulations worldwide, owing to its ability to deliver large doses (up to 1,000 mg) in a short infusion with a favourable safety profile. However, Wolf et al. first highlighted that hypophosphataemia occurs far more frequently with ferric carboxymaltose than with other intravenous preparations [1].

Mechanism and Risk Factors

The underlying pathophysiology relates to FGF23. Normally, iron deficiency increases FGF23 transcription but enhances its proteolytic cleavage, resulting in low levels of intact hormone. Wolf et al. showed that ferric carboxymaltose impairs this cleavage, leading to a sharp rise in intact FGF23 and consequent renal phosphate wasting [4]. This also reduces intestinal absorption via suppression of 1α-hydroxylase activity. Regulatory authorities such as the Medicines and Healthcare products Regulatory Agency (MHRA) have highlighted this mechanism as clinically relevant in recent safety updates [5].

Risk factors include low baseline phosphate, vitamin D deficiency, high-dose or repeated ferric carboxymaltose administration, gastrointestinal malabsorption, and concomitant proton pump inhibitor use. Comparative data suggest that ferric derisomaltose causes hypophosphataemia far less frequently than ferric carboxymaltose [6]. As reviewed by Zoller et al., this complication is increasingly recognised as an important clinical problem, with these risk factors compounding the likelihood [7]. Wolf et al. further demonstrated in randomised trials that the risk of hypophosphataemia is significantly higher with ferric carboxymaltose than with ferric derisomaltose or iron sucrose [8]. In our patient, borderline vitamin D insufficiency, high-dose PPI therapy, and the administration of two 1,000 mg doses within one week likely contributed.

Incidence and Severity

In a randomised controlled trial, Wolf et al. reported that hypophosphataemia occurred in nearly 70% of patients treated with ferric carboxymaltose, compared with fewer than 10% treated with ferric derisomaltose or iron sucrose [8]. Most cases are mild and self-limiting, but severe hypophosphataemia (<0.3 mmol/L) requiring intravenous replacement has been described in only a minority of patients. A comprehensive review by Bartko et al. emphasised the need for monitoring, particularly in patients at higher risk [9]. Case reports by Schaefer et al. and Schouten et al. have documented osteomalacia and fragility fractures following repeated ferric carboxymaltose exposure, underscoring potential long-term risks [3,10]. Although our patient remained clinically asymptomatic beyond fatigue, the prolonged hypophosphataemia is consistent with the findings of Schaefer et al., who described the importance of follow-up monitoring [11].

Service and Systems Impact

Perhaps most striking in this case was the service-level consequence. The patient was otherwise medically fit for discharge after management of his gastrointestinal bleeding, but refractory hypophosphataemia prolonged his admission by eight days. This required multiple cannulations, ultimately central venous access, repeated blood tests, and daily monitoring. Schaefer et al. similarly highlighted the inpatient burden of ferric carboxymaltose-induced hypophosphataemia, even in the absence of overt symptoms [1].

Implications for Practice

Our case supports the need for closer monitoring, particularly in patients with risk factors or where repeated/high doses are used. Awareness of this complication is vital not only for safe prescribing but also for efficient discharge planning. Several trials, including those by Wolf et al. and Schouten et al., have shown that ferric derisomaltose is associated with far lower rates of hypophosphataemia compared with ferric carboxymaltose [1,6,10,11]. Recent narrative reviews have further outlined management strategies and reinforced the need for ongoing vigilance [12]. While agent selection is often influenced by cost and availability, recognition of the differential risk is essential. In our patient, switching to ferric derisomaltose provided a safer option for ongoing iron repletion while avoiding further phosphate depletion.

## Conclusions

This case illustrates that ferric carboxymaltose can lead to severe, prolonged hypophosphataemia that significantly impacts patient care and discharge planning. Although often considered a safe and convenient intravenous iron therapy, its effect on phosphate homeostasis can be clinically meaningful, even in younger patients without renal impairment. In this case, borderline vitamin D insufficiency, high-dose proton pump inhibitor use, and rapid repeat dosing likely contributed to the severity of phosphate depletion. The prolonged inpatient stay, repeated intravenous supplementation, and need for central venous access highlight the wider service implications of this biochemical complication. Switching to ferric derisomaltose allowed continued iron repletion while reducing the risk of further phosphate loss. This case emphasises the importance of recognising risk factors, monitoring serum phosphate after treatment, and considering alternative intravenous iron formulations to prevent avoidable delays in recovery and hospital discharge.
